# Supplementary figures and images for: Gaze-dependent evidence accumulation predicts multi-alternative risky choice behaviour
Source: PLoS Comput Biol. 2022 Jul 6;18(7):e1010283. doi: 10.1371/journal.pcbi.1010283 (PMC9292127; doi:10.1371/journal.pcbi.1010283)

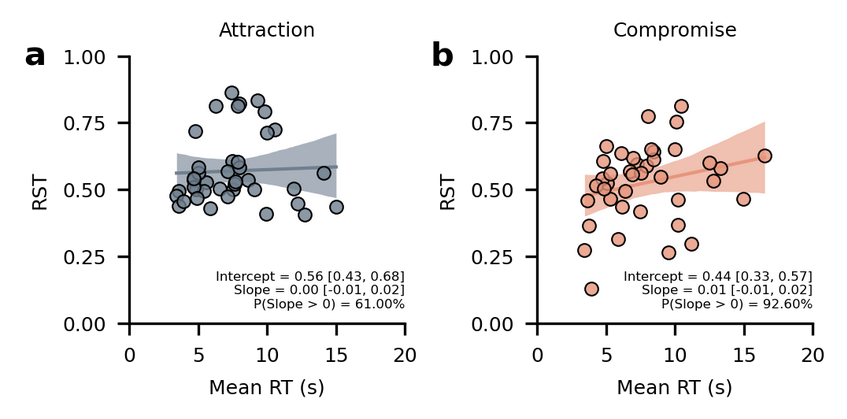

Supplement: S1 Fig — Associations of individual mean response times and context effect strength (RST) in (a) attraction and (b) compromise trials. Statistical annotation denotes mean intercept and slope with associated HDI95 of Bayesian linear regression analyses. (TIFF) [file pcbi.1010283.s001.tiff]

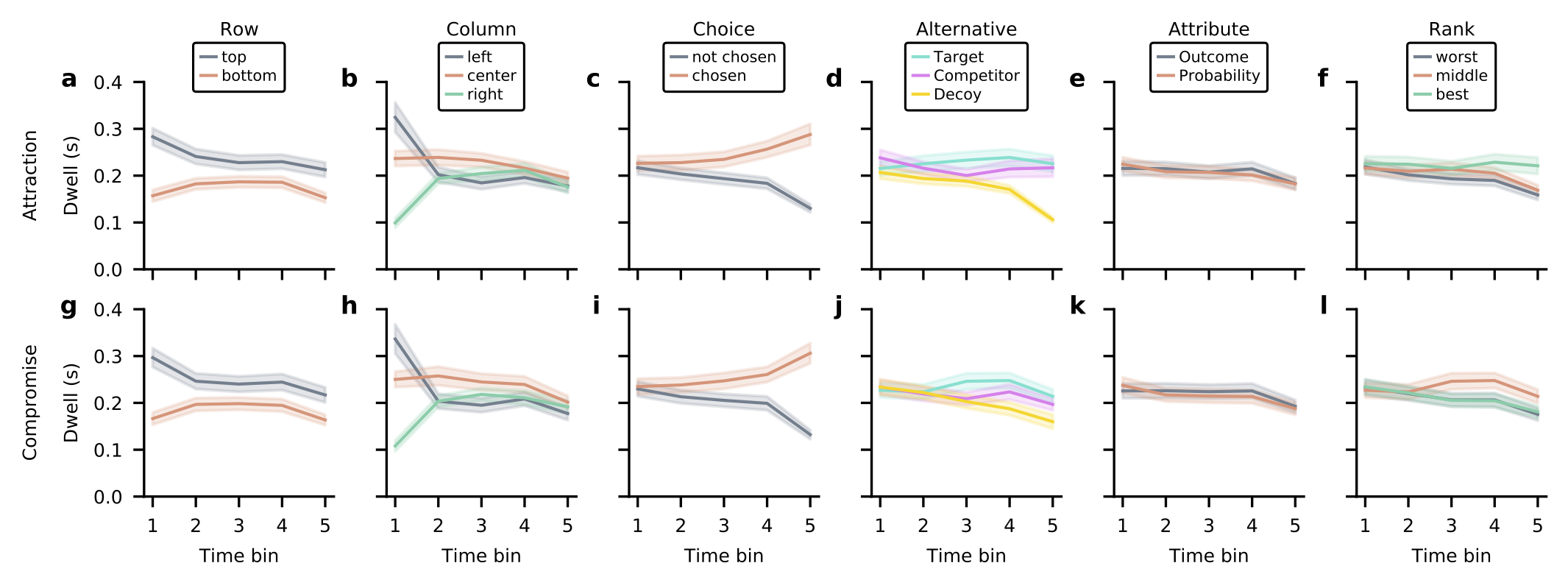

Supplement: S2 Fig — Distribution of gaze over the course of the trial depending on stimulus characteristics. Each panel shows the average dwell time towards AOIs for a given stimulus feature (e.g., horizontal and vertical position) across five time-bins. Data is shown separately for attraction (a-f) and compromise (g-l) trials. (TIFF) [file pcbi.1010283.s002.tiff]

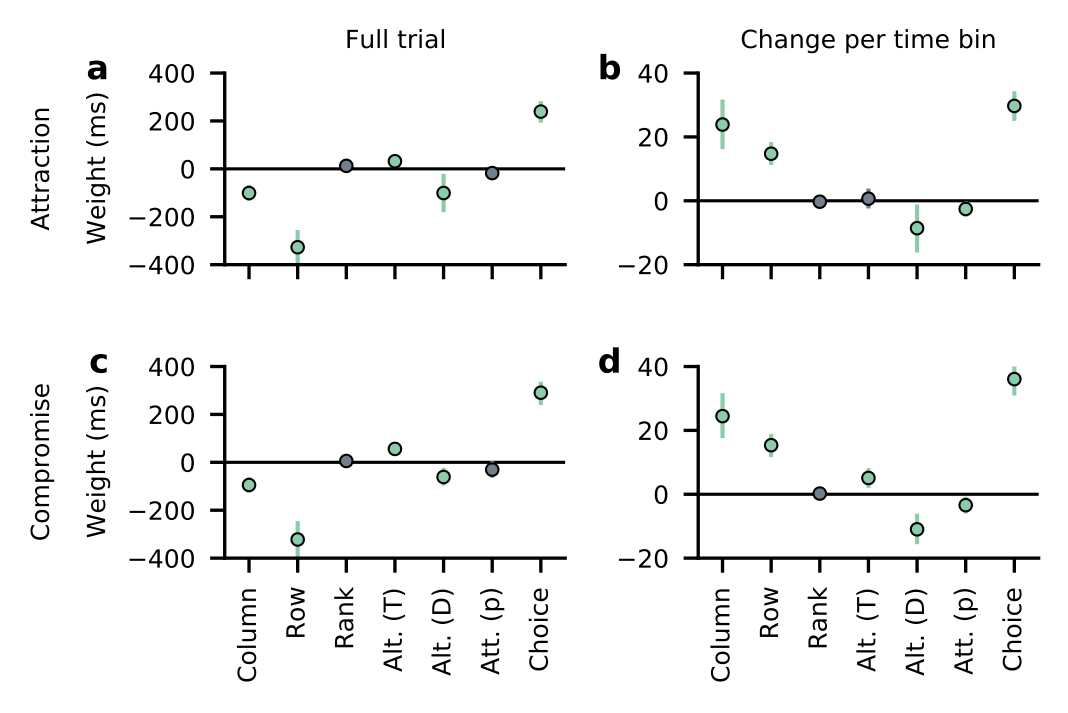

Supplement: S3 Fig — We performed two mixed-effects regression analyses of dwell time towards each AOI onto stimulus properties: (a, c) Regressing the total dwell time towards an AOI over a full trial onto AOI column, row, attribute rank (best, middle or worst value on the attribute), two dummy predictors coding alternative, attribute (probability or outcome) and whether the AOI belonged to the subsequently chosen alternative. (b, d) Second, we binned dwell times in each trial into five time-bins and added an interaction term with time-bin for each predictor. The panels show the interaction term weights. Analyses were carried out separately for attraction (a, b) and compromise (c, d) trials. Regression models had random intercepts and slopes across participants. Points and intervals mark posterior mean estimates and associated HDI95 (coloured green if the interval excluded 0). (TIFF) [file pcbi.1010283.s003.tiff]

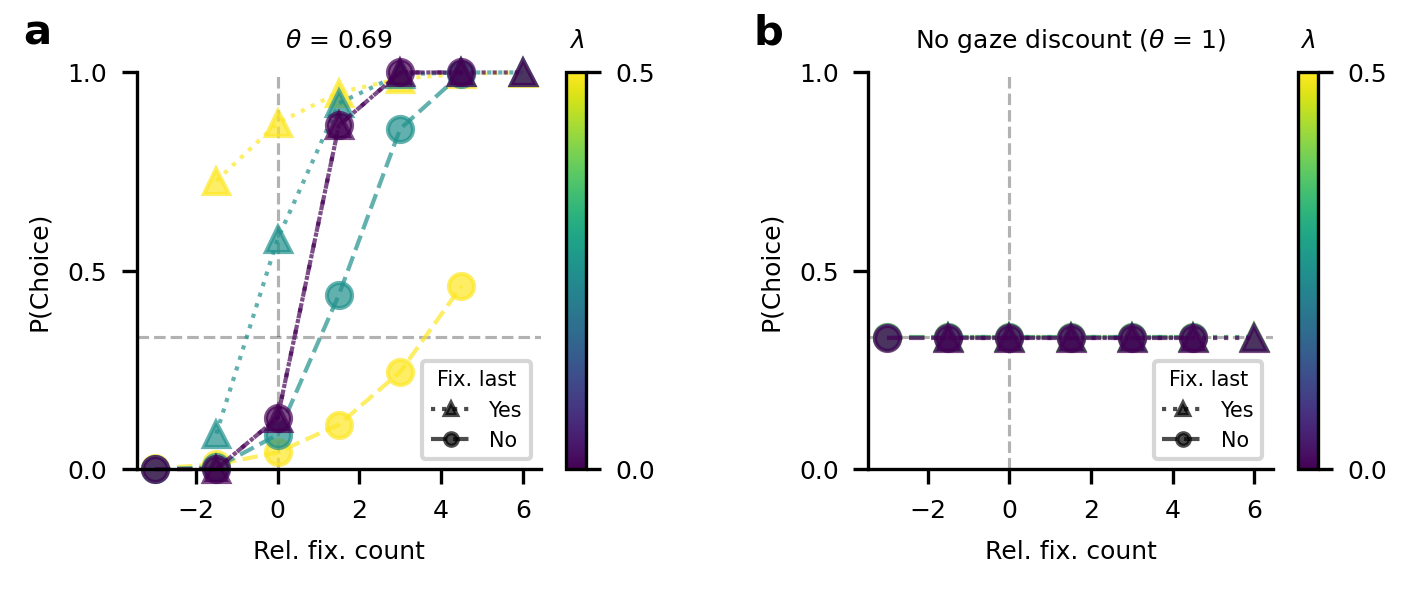

Supplement: S4 Fig — (a) Model-predicted choice probabilities for an item depending on its relative fixation count and whether it was fixated last or not. Different colours refer to different strengths of accumulation leak λ. (b). Like a but without gaze-discount. Predictions for different values of λ fully overlap. All predictions are based on mean empirically estimated model parameters (except for leak parameters λ and the discount parameter θ in b) of trials with three alternatives with equal expected subjective value and all possible sequences of 6 fixations. Horizontal dashed lines represent chance level. See S2 Note for details on the simulation analysis. (TIFF) [file pcbi.1010283.s004.tiff]

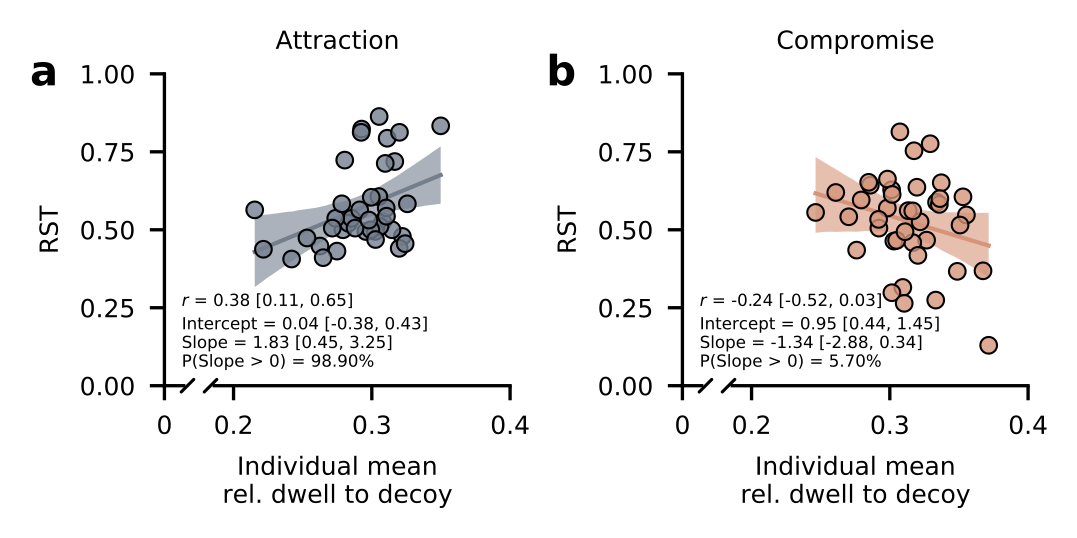

Supplement: S5 Fig — Bayesian linear regressions and correlation analyses of individual mean relative dwell time towards decoy alternatives and context effect strength (RST) in attraction (a) and compromise trials (b). P(Slope > 0) denotes the posterior probability that the regression slope is larger than zero. (TIFF) [file pcbi.1010283.s005.tiff]

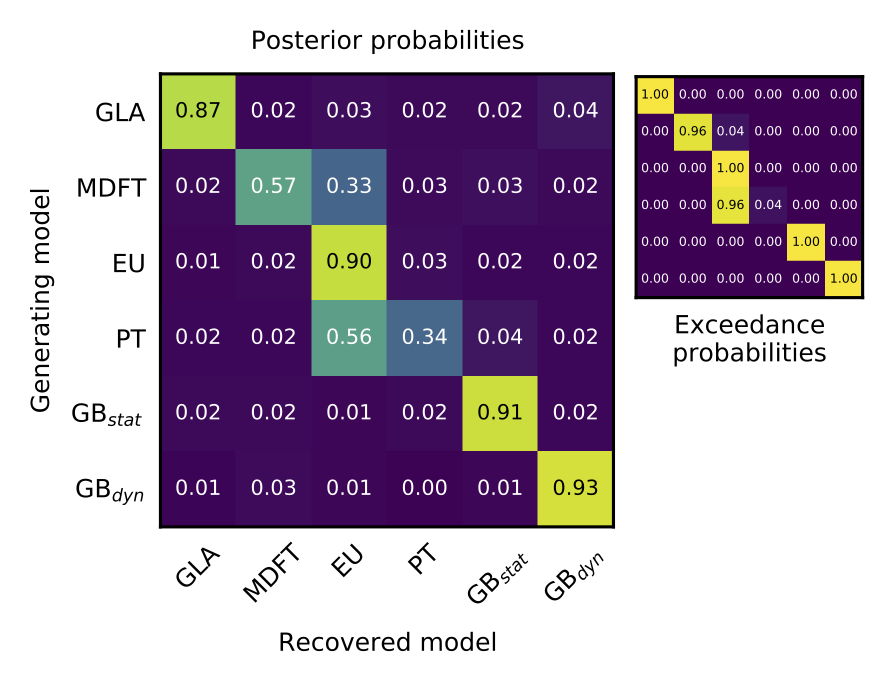

Supplement: S7 Fig — The large panel depicts a confusion matrix summarizing results of a model recovery analysis. Each cell refers to the posterior model probability of a fitted model (column) for a given generating model (row). Perfect recovery would be given by only values of 1 on the diagonal. The smaller matrix depicts exceedance probabilities for data from each generating model (row). Data generated from Prospect Theory (PT) was falsely attributed to Expected Utility Theory (EU). (TIFF) [file pcbi.1010283.s007.tiff]

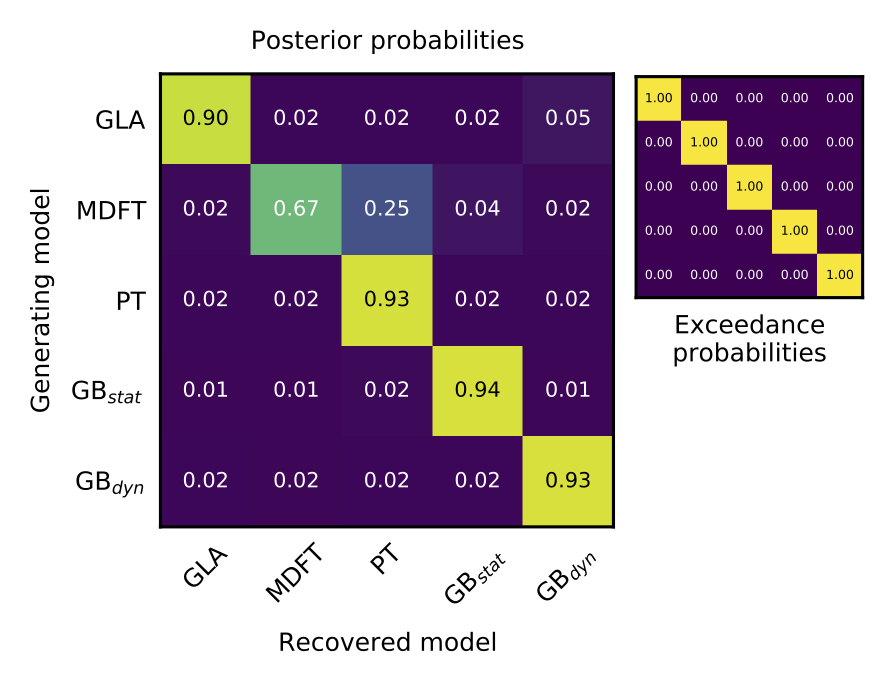

Supplement: S8 Fig — The large panel depicts a confusion matrix summarizing results of a model recovery analysis. Each cell refers to the posterior model probability of a fitted model (column) for a given generating model (row). Perfect recovery would be given by only values of 1 on the diagonal. The smaller matrix depicts exceedance probabilities for data from each generating model (row). (TIFF) [file pcbi.1010283.s008.tiff]

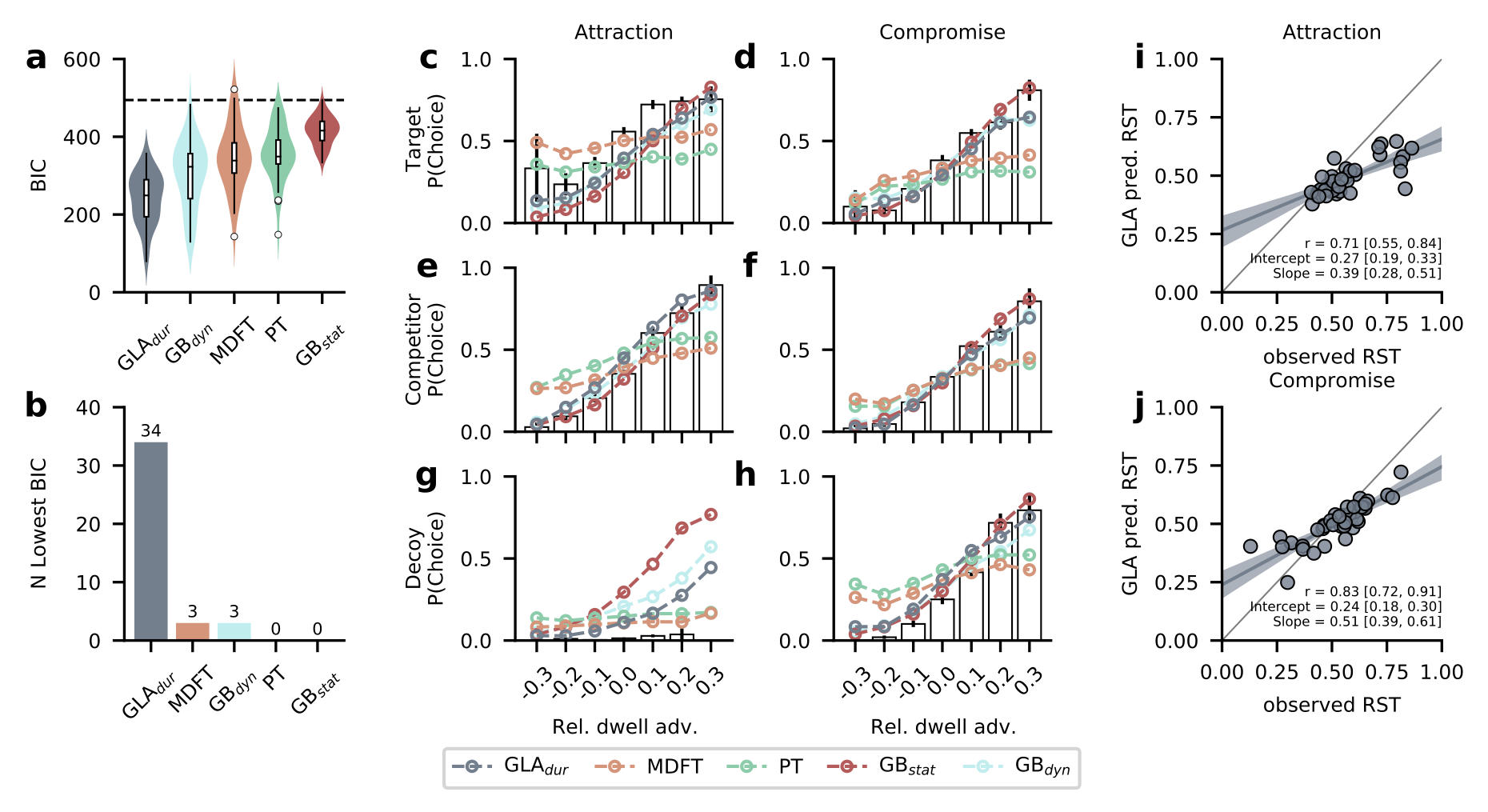

Supplement: S9 Fig — Analog to main text Fig 3. (TIFF) [file pcbi.1010283.s009.tiff]

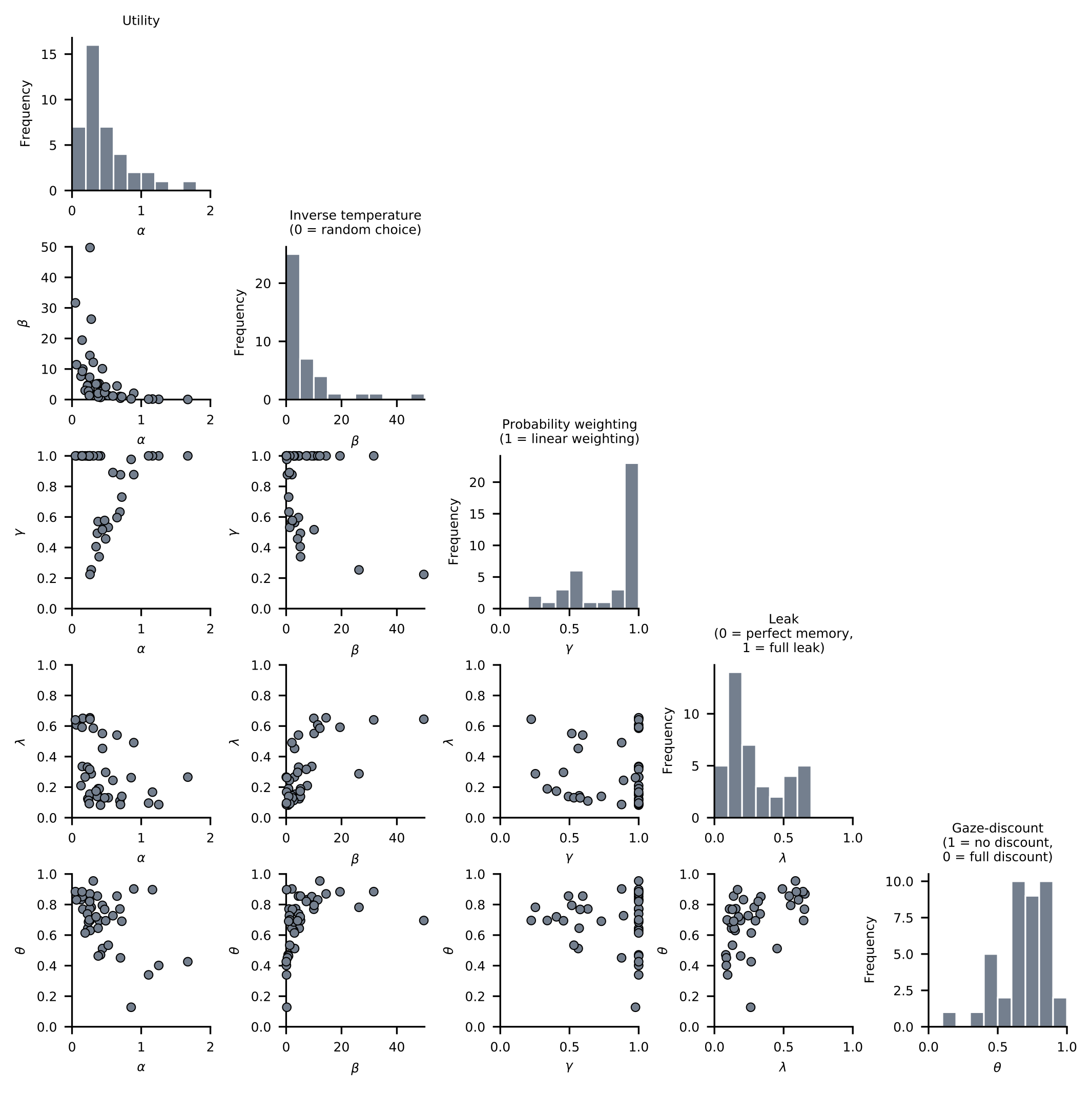

Supplement: S10 Fig — α is the utility parameter. β is the inverse temperature parameter of the choice rule (0 = random choice). γ is the probability weighting parameter (1 = linear weighting). λ is the leak parameter (0 = perfect memory, 1 = full leak of all previous information). θ is the gaze-discount parameter (1 = no gaze-discount, 0 = maximum gaze-discount). (TIFF) [file pcbi.1010283.s010.tiff]

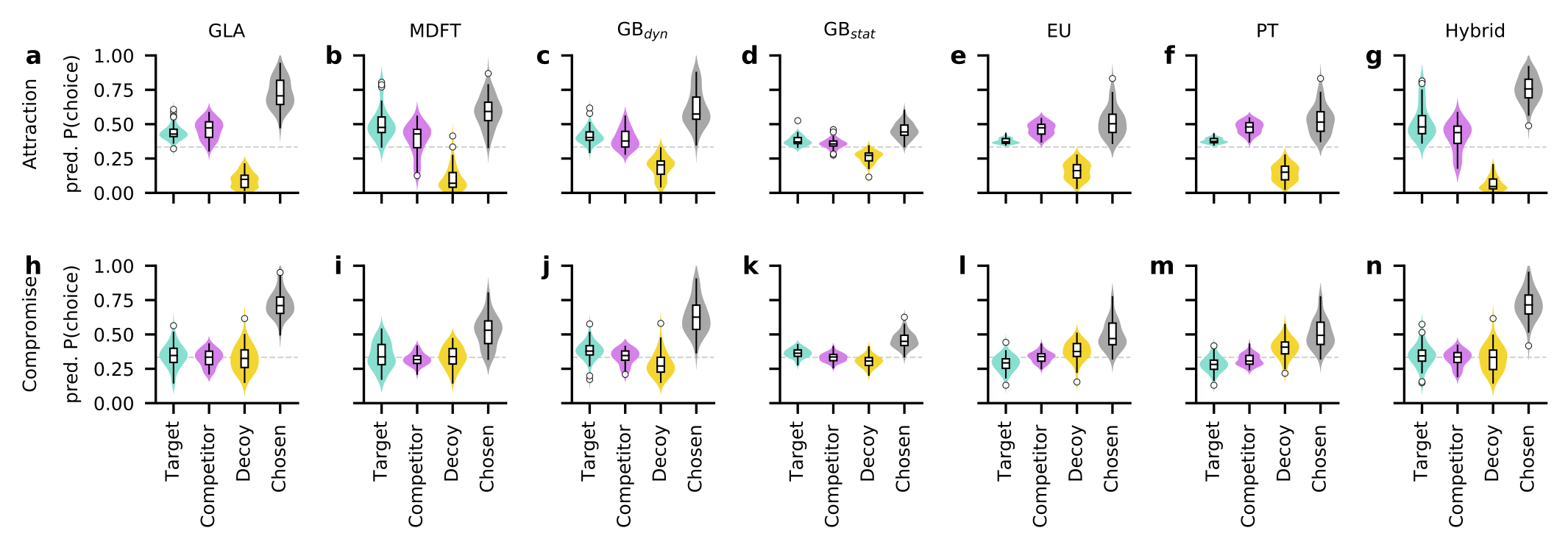

Supplement: S11 Fig — Each panel shows distributions of participant-level mean model-predicted choice probabilities for the target, competitor, decoy and ultimately chosen alternative. Predictions for attraction and compromise trials are displayed separately in the top (a-g) and bottom rows (h-n). Predictions were computed using individual maximum likelihood estimates. The hybrid model (g, n) was derived from the switchboard analysis and combines an alternative-wise gaze-discount with a distance-dependent inhibition mechanism. Violin plots show kernel density estimates of distributions of individual values. Box plots mark lower and upper quartiles and median. Whiskers extend from first and last datum within 1.5 times the interquartile range from lower and upper quartiles, respectively. Values outside this range are indicated by open circles. (TIFF) [file pcbi.1010283.s011.tiff]

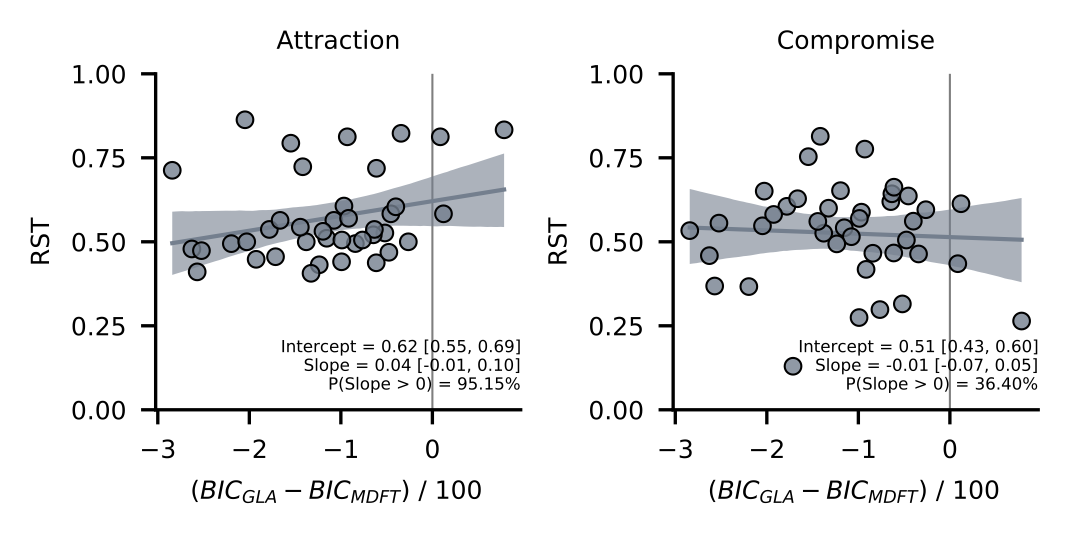

Supplement: S12 Fig — Relative model fits of MDFT (indicated by BIC difference between GLA and MDFT) tended to be higher for participants with higher RST in attraction trials (left panel; slope = 0.04, HDI95 = [-0.01, 0.09] increase in RST per 100 unit increase in BIC difference, 93.6% of posterior mass above 0), but not compromise trials (right panel), even though 7 out of 9 participants with attraction RST above 0.7 were better described by GLA overall (participants left of dashed vertical line). (TIFF) [file pcbi.1010283.s012.tiff]

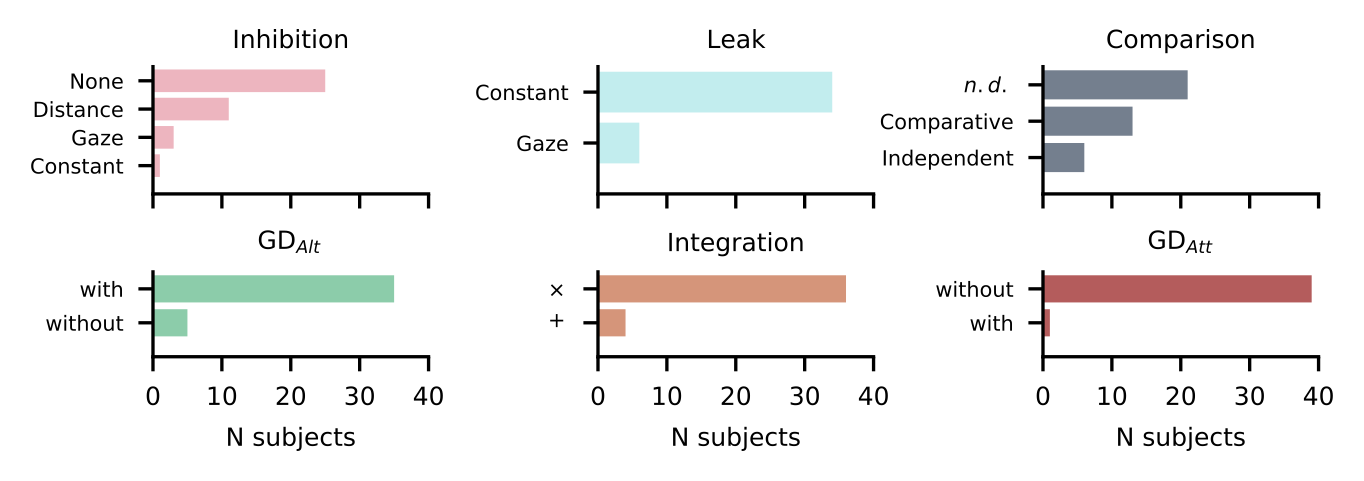

Supplement: S13 Fig — Most participants were best described by model variants that included multiplicative attribute integration, with alternative-wise gaze discount, no attribute-wise gaze discount, constant leakage and no inhibition. (TIFF) [file pcbi.1010283.s013.tiff]

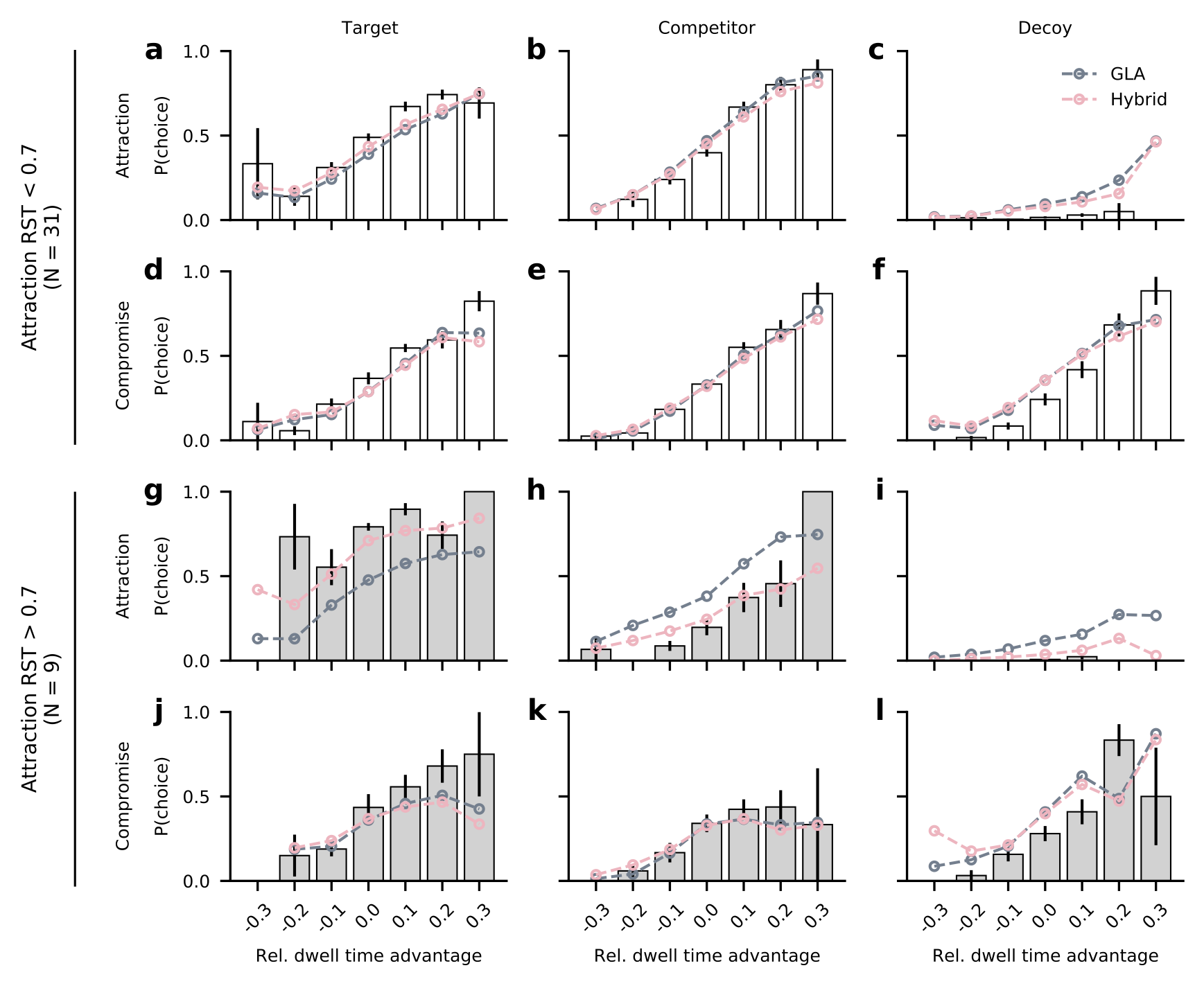

Supplement: S14 Fig — (a-f) Data and model predictions for participants with weaker attraction effects (RST < 0.7). (g-l) Data and model predictions for participants with strong attraction effects (RST > 0.7) Each column refers to one choice alternative: Target (first column; a, d, g, j); Competitor (second column; b, e, h, k); Decoy (third column; c, f, i, l). Rows refer to trials in attraction (a-c, g-i) and compromise trials (d-f, j-l). White and grey bars and error bars show observed mean ± s.e. choice probabilities computed from even-numbered trials, for participants with weaker and stronger attraction effects, respectively. Coloured lines indicate model predictions derived from 50 simulations for each odd-numbered trial. (TIFF) [file pcbi.1010283.s014.tiff]

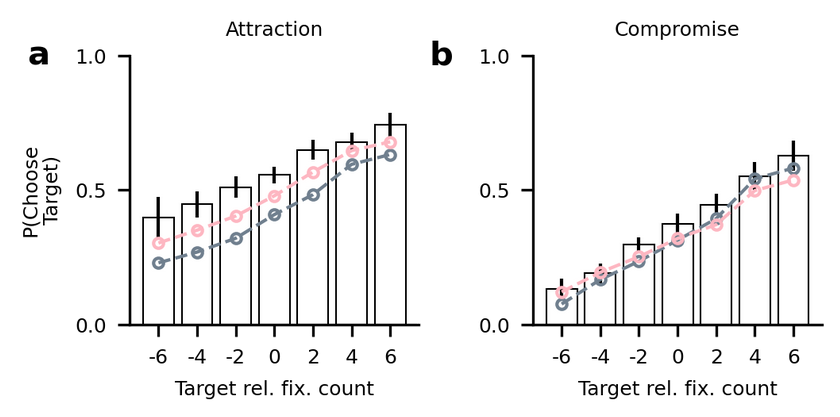

Supplement: S15 Fig — Analog to main text Fig 5E and 5F. Pink and gray lines refer to the Hybrid and GLA models, respectively. (TIFF) [file pcbi.1010283.s015.tiff]
