# Supplementary material for: Gaze-dependent evidence accumulation predicts multi-alternative risky choice behaviour
Source: PLoS Comput Biol. 2022 Jul 6;18(7):e1010283. doi: 10.1371/journal.pcbi.1010283 (PMC9292127; doi:10.1371/journal.pcbi.1010283)
Supplement: S1 Note — (DOCX) [file pcbi.1010283.s016.docx]

## S1 Note

**Regression analyses of gaze behaviour** We performed two linear mixed effects regressions of total dwell time towards an AOI in each trial, separate for attraction and compromise trials (S2 Fig.). In the first ("full trial") model, the dependent variable was total dwell time towards an AOI across the full trial. This model used the following predictors: Vertical position (row, centred: -0.5 = top row, +0.5, bottom row), horizontal position (column, centred: -1 = left, 0 = centre, +1 = right), attribute (dummy coding probability attribute $p$), within dimension attribute rank (centred, -1 = worst, 0 = intermediate, +1 = best on attribute), target (dummy coded), decoy (dummy coded) and a dummy coded predictor for the ultimately chosen alternative. For the second model, we partitioned the dwell-data into five equally sized time-bins. The dependent variable in this model then was total dwell time towards an AOI within a time-bin. This model included the same predictors as the "full trial" model. Crucially, it also included interaction terms for each predictor with the time-bin variable, and time-bin as additional predictor. Both models included random intercepts and slopes for each participant. Bayesian posterior distributions of the regression weights were estimated using the bambi Python library [1], with default priors [2], sampling four chains with 2000 samples each, after a tuning phase of 500 samples. Convergence was diagnosed visually and by means of the Gelman-Rubin statistic ($|1-\hat{R}|\leq0.05$ for all chains).

Regression weight estimates are shown in S3 Fig. Across the trial, we find strong effects of position on dwell time, so that dwell times towards the top and left were longer. Significant negative interaction effects of the row and column predictors with time showed that these effects diminish across the trial. We also find a gaze-cascade effect [3,4], where dwell times to AOIs belonging to the ultimately chosen alternative are longer across the trial (and increasingly so throughout the trial, indicated by the positive interaction term with time). Dwell times to decoys decreased significantly during the trial, and across the full trial, dwell times to decoys were shorter than other alternatives. Similarly, dwell times towards probability attributes *p* shortened across the trial. Across the full trial, however, dwell times towards probability AOIs were not shorter than those to outcome AOIs. Finally, dwell times to target alternatives were longer across the trial in both compromise and attraction trials. In addition, this effect increased throughout the trial in compromise, but not in attraction trials. Note that these effects are independent of the effect of choice, as choice is a separate predictor in the model. We could not find an association between the attribute rank (being the worst, best, or intermediate item on an attribute) and dwell time.

**Direction of information search** We further analysed participants’ direction of information search. Therefore, we counted the number of vertical (transitions within the same alternative), horizontal (within the same row, between alternatives) and diagonal (between rows and alternatives) transitions. On average, participants made over 7 horizontal transitions in attraction (mean $\pm$ s.d. = 7.28 $\pm$ 2.36) and compromise (mean $\pm$ s.d. = 7.29 $\pm$ 2.63) trials, with no meaningful difference between effects. Participants made, however, more vertical transitions in compromise trials (mean $\pm$ s.d. = 7.55 $\pm$ 3.11) than attraction trials (mean $\pm$ s.d. = 7.17 $\pm$ 3.06; mean difference = 0.39, HDI${}_{95}$ = [0.11, 0.64], $d$ = 0.49, HDI${}_{95}$ = [0.13, 0.82]). The number of diagonal transitions was lower overall, but higher in attraction (mean $\pm$ s.d. = 2.88 $\pm$ 1.18) than compromise trials (mean $\pm$ s.d. = 2.72 $\pm$ 1.31; mean difference = 0.19, HDI${}_{95}$ = [0.06, 0.32], $d$ = 0.62, HDI${}_{95}$ = [0.14, 1.17]).

While vertical transitions always translate to transitions "within alternative", horizontal transitions are not necessarily always "within attribute", since the attribute positions of each alternative were random in the task. We therefore recoded transitions as "within alternative", "within attribute" and "between alternatives and attributes" and computed the Payne Index [5] for each trial as:

$$\begin{aligned} \text{Payne Index}=\frac{N_{\text{within alt.}}-N_{\text{within att.}}}{N_{\text{within alt.}}+N_{\text{within att.}}}\#\left( 1 \right) \end{aligned}$$

A more positive value on the index indicates more processing within alternatives, whereas more negative values indicate more processing between alternatives, within the same attribute dimension. Overall, the average Payne Index was slightly positive for both attraction (mean $\pm$ s.d. = 0.10 $\pm$ 0.16) and compromise trials (mean $\pm$ s.d. = 0.14 $\pm$ 0.18), suggesting a mixture of within-alternative and within-attribute processing, with slightly more processing within alternatives. It was, however, lower in attraction trials (mean difference = 0.04, HDI${}_{95}$ = [0.01, 0.06], $d$ = 0.51, HDI${}_{95}$ = [0.16, 0.87]), implying comparably more processing between alternatives in attraction trials.

We also analyzed transition counts with respect to target, competitor, and decoy alternatives. Replicating prior work [6], we found that participants made more transitions between target and decoy options than between competitor and decoy options in attraction (mean difference = 0.99, HDI${}_{95}$ = [0.75, 1.24], $d$ = 1.39, HDI${}_{95}$ = [0.93, 1.90]) and compromise trials (mean difference = 0.61, HDI${}_{95}$ = [0.41, 0.80], $d$ = 1.31, HDI${}_{95}$ = [0.70, 2.03]).

## References

1. Capretto T, Piho C, Kumar R, Westfall J, Yarkoni T, Martin OA. Bambi: A simple interface for fitting Bayesian linear models in Python. arXiv:201210754 [stat]. 2021 [cited 27 May 2021]. Available: http://arxiv.org/abs/2012.10754

2. Westfall J. Statistical details of the default priors in the Bambi library. arXiv:170201201 [stat]. 2017 [cited 3 Feb 2020]. Available: http://arxiv.org/abs/1702.01201

3. Shimojo S, Simion C, Shimojo E, Scheier C. Gaze bias both reflects and influences preference. Nat Neurosci. 2003;6: 1317–1322. doi:10.1038/nn1150

4. Mullett TL, Stewart N. Implications of visual attention phenomena for models of preferential choice. Decision. 2016;3: 231. doi:10.1037/dec0000049

5. Payne JW. Task complexity and contingent processing in decision making: An information search and protocol analysis. Organizational Behavior and Human Performance. 1976;16: 366–387. doi:10.1016/0030-5073(76)90022-2

6. Marini M, Ansani A, Paglieri F. Attraction comes from many sources: Attentional and comparative processes in decoy eﬀects. Judgment and Decision Making. 2020;15: 23.
