# Supplementary material for: Gaze-dependent evidence accumulation predicts multi-alternative risky choice behaviour
Source: PLoS Comput Biol. 2022 Jul 6;18(7):e1010283. doi: 10.1371/journal.pcbi.1010283 (PMC9292127; doi:10.1371/journal.pcbi.1010283)
Supplement: S2 Note — (DOCX) [file pcbi.1010283.s017.docx]

**S2 Note**

While GLA’s leak mechanism is *constant* (i.e., the same rate of decay is applied to all accumulator values, irrespective of gaze allocation), its combined use with a gaze-dependent mechanism (i.e., the gaze discount) yields distinct gaze-dependent predictions of choice behaviour (S4 Fig.). To illustrate this, we performed the following simulation procedure: First, we created three choice alternatives with equal subjective expected value according to a GLA model using mean empirical parameter estimates (see S1 Table). Next, we computed the model’s choice probabilities for each possible sequence of 6 fixated alternatives (e.g., “Alt. 1, Alt. 2, Alt. 3, Alt. 1, Alt. 2, Alt. 3”). Finally, we computed the average predicted choice probability for the Alternative 1, as a function of its relative fixation count (number of fixations towards it minus the mean number of fixations towards the other two alternatives) and whether it was the last fixated alternative in the sequence or not. For illustration of the leak parameter λ’s effect, we repeated this procedure for λ values of 0, 0.25 and 0.5. S4a Fig. shows the simulation results. Notably, increasing values of λ allow the model to predict more diverging choice probabilities for alternatives fixated last or not.

Therefore, even if the leak itself does not depend on gaze allocation, it allows the model to use choice-relevant information contained in the temporal order of fixations*.* This, however, is only the case when the model also uses a gaze discount: we repeated the analysis setting the gaze-discount parameter θ to 1 (no discounting; S4b Fig.). Now, the model’s predictions are not affected by relative fixation counts or the last fixation target.
