# Supplementary material for: Gaze-dependent evidence accumulation predicts multi-alternative risky choice behaviour
Source: PLoS Comput Biol. 2022 Jul 6;18(7):e1010283. doi: 10.1371/journal.pcbi.1010283 (PMC9292127; doi:10.1371/journal.pcbi.1010283)
Supplement: S3 Note — (DOCX) [file pcbi.1010283.s018.docx]

## S3 Note

**No process evidence that strong attraction responders follow simple choice rule** Using process measures, we performed multiple tests of the hypothesis, that individuals with strong attraction effects follow a simple choice rule of choosing the dominant alternative. First, we tested whether the strength of individual attraction effects (individual RST in attraction trials) was related to differences in mean response times (RTs) in attraction trials. If individuals used a choice rule, their choices might be made faster, as they do not engage in multiple pairwise comparisons or calculations of expected outcomes. There was no correlation between the two measures ($r$ = 0.06, HDI${}_{95}$ = [-0.24, 0.34]). Similarly, no relationship was found between individual RST and the number of fixations in attraction trials ($r$ = 0.06, HDI${}_{95}$ = [-0.25, 0.35]). Mean RTs in attraction trials did not meaningfully differ between trials with target choices and trials with other choices ($d$ = -0.2, HDI${}_{95}$ = [-0.67, 0.25]). Next, we tested whether individuals with strong attraction effects committed to a choice once they learned about the dominance relationship in the stimuli, as if using the dominance relationship as a stopping rule, or if they kept exploring the stimuli. There was, however, no relationship between individual RST and the mean number of fixations after all target and decoy attributes were fixated at least once ($r$ = 0.04, HDI${}_{95}$ = [-0.28, 0.31]). The same analysis using fixation counts after target and decoy alternatives were both seen at least once on any attribute revealed no effect either ($r$ = 0.11, HDI${}_{95}$ = [-0.20, 0.40]). Taken together, we did not find any evidence based on process data to support the hypothesis that strong attraction responders used a simple choice rule.
