# Supplementary material for: Gaze-dependent evidence accumulation predicts multi-alternative risky choice behaviour
Source: PLoS Comput Biol. 2022 Jul 6;18(7):e1010283. doi: 10.1371/journal.pcbi.1010283 (PMC9292127; doi:10.1371/journal.pcbi.1010283)
Supplement: S1 Table — α is the utility parameter. β is the inverse temperature parameter of the choice rule (0 = random choice). γ is the probability weighting parameter (1 = objective probability weighting). λ is the leak parameter (0 = perfect memory, 1 = full leak of all previous information). θ is the gaze-discount parameter (1 = no gaze-discount, 0 = maximum gaze-discount). (DOCX) [file pcbi.1010283.s019.docx]

|  | mean | SD | min | 25% | 50% | 75% | max |
| --- | --- | --- | --- | --- | --- | --- | --- |
| $\alpha$ | 0.47 | 0.35 | 0.05 | 0.25 | 0.37 | 0.61 | 1.67 |
| $\beta$ | 6.81 | 9.87 | 0.04 | 1.13 | 3.25 | 8.04 | 49.74 |
| $\gamma$ | 0.81 | 0.25 | 0.22 | 0.58 | 1.0 | 1.0 | 1.0 |
| $\lambda$ | 0.29 | 0.2 | 0.08 | 0.14 | 0.23 | 0.46 | 0.65 |
| $\theta$ | 0.69 | 0.18 | 0.13 | 0.63 | 0.72 | 0.83 | 0.95 |

S1 Table: **Summary of GLA estimates.** $\alpha$ is the utility parameter. $\beta$ is the inverse temperature parameter of the choice rule (0 = random choice). $\gamma$ is the probability weighting parameter (1 = objective probability weighting). $\lambda$ is the leak parameter (0 = perfect memory, 1 = full leak of all previous information). $\theta$ is the gaze-discount parameter (1 = no gaze-discount, 0 = maximum gaze-discount).
