# Supplementary material for: Gaze-dependent evidence accumulation predicts multi-alternative risky choice behaviour
Source: PLoS Comput Biol. 2022 Jul 6;18(7):e1010283. doi: 10.1371/journal.pcbi.1010283 (PMC9292127; doi:10.1371/journal.pcbi.1010283)
Supplement: S2 Table — Switch levels that depend on gaze-data are shaded blue. Note that due to the model fitting procedure, where model predicted choice probabilities are derived from a soft-max function over the final accumulator values, the comparison switch levels independent and comparative are not distinguishable for a subset of the model space, reducing the total number of unique variants to 160. (DOCX) [file pcbi.1010283.s020.docx]

| **Switch** | **Gaze-independent levels** | | | | **Gaze-dependent levels** | | **N** |
| --- | --- | --- | --- | --- | --- | --- | --- |
| **Attribute integration** | **× Multiplicative** Outcomes and probabilities combine multiplicatively into expected utilities [1,2]. | | **+ Weighted additive** Outcomes and probabilities are normalized, weighted and added [3]. | | *n.a.* | | 2 |
| **Comparison** | **Independent** Accumulation of absolute item values per alternative [2,4]. | | **Comparative** Accumulation of relative values per alternative [5]. | | *n.a.* | | 2 |
| **Attribute-wise gaze-discount** | **False** Fixated and non-fixated alternatives are processed equally. | | | | **True** Non-fixated alternatives’ values are discounted by a parameter θ [6]. | | 2 |
| **Alternative-wise gaze-discount** | **False** Fixated and non-fixated attributes are processed equally. | | | | **True** Attributes on the non-fixated dimension are discounted by a parameter η [7,8]. | | 2 |
| **Accumulation leak** | **None** Perfect integration over fixations. | | **Constant** With each fixation, accumulators leak information proportional to their current value, controlled by parameter λ [5,9]. | | **Gaze-dependent** With each fixation, accumulators of non-fixated alternatives leak information, as in Constant leak [10]. | | 3 |
| **Accumulator inhibition** | **None** No inhibition between accumulators. | **Constant** Accumulators inhibit each other proportional to their current value, controlled by parameter φ [11]. | | **Distance-dependent** Inhibition between accumulators depends on pairwise distance between alternatives in attribute space. Parameters *w_d_* and φ [5]. | | **Gaze-dependent** With each fixation, accumulators of non-fixated alternatives are inhibited, proportional to the currently fixated items’ accumulator value. Controlled by parameter φ. | 4 |
| **Total variants** |  | | | | | | 192 |

**Supplementary Table 2. Overview of the nodes and switch-levels used in the switchboard analysis.** Switch levels that depend on gaze-data are shaded blue. Note that due to the model ﬁtting procedure, where model predicted choice probabilities are derived from a soft-max function over the ﬁnal accumulator values, the comparison switch levels independent and comparative are not distinguishable for a subset of the model space, reducing the total number of unique variants to 160.

**References**

1. Tversky A, Kahneman D. Advances in prospect theory: Cumulative representation of uncertainty. J Risk Uncertainty. 1992;5: 297–323. doi:10.1007/BF00122574

2. Glickman M, Sharoni O, Levy DJ, Niebur E, Stuphorn V, Usher M. The formation of preference in risky choice. PLOS Computational Biology. 2019;15: e1007201. doi:10.1371/journal.pcbi.1007201

3. Rouault M, Drugowitsch J, Koechlin E. Prefrontal mechanisms combining rewards and beliefs in human decision-making. Nat Commun. 2019;10: 1–16. doi:10.1038/s41467-018-08121-w

4. Bhatia S. Associations and the accumulation of preference. Psychological Review. 2013;120: 522–543. doi:10.1037/a0032457

5. Roe RM, Busemeyer JR, Townsend JT. Multialternative decision field theory: A dynamic connectionst model of decision making. Psychological Review. 2001;108: 370–392. doi:10.1037/0033-295X.108.2.370

6. Krajbich I, Armel C, Rangel A. Visual fixations and the computation and comparison of value in simple choice. Nat Neurosci. 2010;13: 1292–1298. doi:10.1038/nn.2635

7. Krajbich I, Lu D, Camerer C, Rangel A. The attentional drift-diffusion model extends to simple purchasing decisions. Front Psychol. 2012;3: 193. doi:10.3389/fpsyg.2012.00193

8. Fisher G. An attentional drift diffusion model over binary-attribute choice. Cognition. 2017;168: 34–45. doi:10.1016/j.cognition.2017.06.007

9. Usher M, McClelland JL. The time course of perceptual choice: The leaky, competing accumulator model. Psychological Review. 2001;108: 550–592. doi:10.1037/0033-295X.108.3.550

10. Ashby NJS, Jekel M, Dickert S, Glöckner A. Finding the right fit: A comparison of process assumptions underlying popular drift-diffusion models. Journal of Experimental Psychology: Learning, Memory, and Cognition. 2016;42: 1982–1993. doi:10.1037/xlm0000279

11. Usher M, McClelland JL. Loss Aversion and Inhibition in Dynamical Models of Multialternative Choice. Psychological Review. 2004;111: 757–769. doi:10.1037/0033-295X.111.3.757
