# Supplementary material for: Gaze-dependent evidence accumulation predicts multi-alternative risky choice behaviour
Source: PLoS Comput Biol. 2022 Jul 6;18(7):e1010283. doi: 10.1371/journal.pcbi.1010283 (PMC9292127; doi:10.1371/journal.pcbi.1010283)
Supplement: S3 Table — All ten model variants that fit the data best on average used some form of gaze-dependence (blue shaded cells), mostly an alternative-wise gaze discount. "n.d." denotes variants where comparison mechanisms were not distinguishable by the analysis. (DOCX) [file pcbi.1010283.s021.docx]

| **Rank** | $\mathbf{G}\mathbf{D}_{\mathbf{Alt}}$ | $\mathbf{G}\mathbf{D}_{\mathbf{Att}}$ | **Leak** | **Inhibition** | **Integration** | **Comparison** | **BIC** |
| --- | --- | --- | --- | --- | --- | --- | --- |
| 1 | **Yes** | No | Constant | None | Multiplicative | *n.d.* | 232.08 |
| 2 | No | No | Constant | **Gaze** | Multiplicative | Independent | 235.94 |
| 3 | **Yes** | No | Constant | **Gaze** | Multiplicative | Comparative | 236.75 |
| 4 | **Yes** | **Yes** | Constant | None | Multiplicative | *n.d.* | 237.21 |
| 5 | **Yes** | No | Constant | **Gaze** | Multiplicative | Independent | 237.31 |
| 6 | **Yes** | No | Constant | Constant | Multiplicative | *n.d.* | 237.40 |
| 7 | **Yes** | No | **Gaze** | None | Multiplicative | Comparative | 238.41 |
| 8 | No | **Yes** | Constant | **Gaze** | Multiplicative | Independent | 241.04 |
| 9 | **Yes** | No | Constant | Distance | Multiplicative | Comparative | 241.40 |
| 10 | **Yes** | **Yes** | Constant | **Gaze** | Multiplicative | Comparative | 241.78 |

S3 Table: **Overview of average best fitting model variants.** All ten model variants that fit the data best on average used some form of gaze-dependence (blue shaded cells), mostly an alternative-wise gaze discount. *"n.d."* denotes variants where comparison mechanisms were not distinguishable by the analysis.
