# Supplementary material for: Gaze-dependent evidence accumulation predicts multi-alternative risky choice behaviour
Source: PLoS Comput Biol. 2022 Jul 6;18(7):e1010283. doi: 10.1371/journal.pcbi.1010283 (PMC9292127; doi:10.1371/journal.pcbi.1010283)
Supplement: Attachment — Submitted filename: molter_et-al_gda-context_point-by-point-response.docx [file pcbi.1010283.s022.docx]

# Point-by-point-response

## Reviewer 1

### General remarks

The present work tackles the issue of decoy effects in risky decisions. Various proposals have attempted to explain these violations of independence of irrelevant alternatives (IIA) in multi-alternative scenarios, and recently it has been highlighted that attentional capture processes may be driving them. This work expands this line, presenting a detailed analysis of how visual attention could be involved in attraction and compromise effects. A new behavioral and eye-tracking experiment plus a systematic and comprehensive modeling approach are included in this paper, showing the centrality that gaze may be playing in the appearance of these biases.

I reckon this work may be of interest for the neuroeconomics and decision-making community, adding further support to this alternative view of decoy effects, in addition to being a relevant application of attentional accumulation models. The paper is well written and figures very helpful (special mention to Figure 4 for schematizing the switchboard analysis). I suggest minor clarifications to some points:

### Major points

1. In the introduction you mention three effects (attraction, compromise, and similarity). The results in the paper are focused on attraction and compromise, however, there is not much elaboration on the similarity effect. Please complement or correct this.

We thank the reviewer for pointing us to this disparity. We have added clarifications for focusing on attraction and compromise effects in the introduction (ll. 101ff) and results (ll. 166ff) sections.

2. Given that attention is the factor driving attraction and compromise effects, do you think it makes sense to look for a modulation of the decoy effect by the amount of time the decoy was fixated? It may be interesting to check directly from participant’s behavior, whether (relative) gaze to decoy at individual level modulates individual RST magnitude (i.e. participants that gaze more the decoy have higher RST?). Even more, do you think is it possible to see at a within-participant level that variations in gaze could affect the magnitude of the effect? (e.g. split participant trials for lower and higher decoy gaze time and check if the RST is higher for high decoy gaze time trials). Maybe the relationship between (theta, attention bias parameter) and RST between subjects may also be informative.

We thank the reviewer for these thoughtful suggestions. As suggested, we have conducted multiple additional analyses on the relationships between individuals’ mean dwell times towards decoy alternatives and their context effect strengths, both between and within individuals.

In brief, we find positive associations in attraction trials, suggesting that longer dwell towards dominated decoys is associated with more target choices. In contrast, the association trends negative in compromise trials.

We report the results of these analyses in the results section (ll. 324ff) and added a figure illustrating the associations between individuals’ mean relative dwell towards decoy alternatives and their context effect strengths (Supplementary Fig. 5).

We also tested the association between GLA’s gaze-discount parameter and RST but found no association.

3 . Are participants paying more attention (more relative gaze time) to the decoy in compromise vs attraction trials? Do you think this could be connected to the fact that evidence for the compromise effect was weaker than for attraction effect? I think more elaboration on why compromise effects were modest in this experiment could be added to the discussion. Can you comment on why participants chose the compromise decoy with the higher outcome (Cb) in higher proportion to low value (low risk) decoy (Ca), when it was expected them to be the same? I’m guessing different traces of attention could be expected for these two cases

We thank the reviewer for these suggestions. We have tested and reported differences in relative dwell times towards alternatives between trial types (ll. 309ff). We find that decoys were indeed looked at longer in compromise trials (mean difference = 0.02, HDI_95_ = [0.007, 0.033], mean *d* = 0.53, HDI_95_ = [0.20, 0.90]) which mirrors their choice probabilities; Decoy alternatives were also chosen more frequently in compromise trials. We note, however, that decoy choices by themselves do not affect our measure of context effect strength, which is only computed from target and competitor choices.

We included a section about possible reasons for why observed compromise effects were asymmetric with respect to the decoy type (ll. 250ff). We suspect that the indifference estimation procedure could be one reason for the observed asymmetries.

We also included a more elaborate discussion about the weaker compromise effects in our study (ll. 678ff), relating our results to marginal results from other studies and possible explanations.

4. Please include further details regarding the model fit: is the fitting considering attraction and compromise trials together or they are fitted separately? Are you fitting to odd-numbered trials and testing with even-numbered trials? What was the method employed to extract the fixations for the simulations? What’s the situation for the model fitted in switchboard? Did you fit the two conditions together? Is table 2 estimated from attraction trials only?

We thank the reviewer for pointing us to this lack of detail.

All models were fit to *all* trials of each participant. This included attraction, compromise and filler trials. This was the procedure for the extant models, as well as the switchboard models. Only figures overlaying model-predicted associations of gaze and choice show observed data from even-numbered trials and model predictions for odd-numbered trials, as indicated in the figure legends.

We have added additional details to the parameter estimation and model comparison sections (ll. 1042ff) and the description of the switchboard analysis (ll. 1180ff).

5. Was there a reason to not include a model like GLA but without the accumulation leak? If I understood well, GLA and GBdyn models include a decay term that does not depend on gaze, so it may be raised that gaze AND accumulation decay are equally relevant to capture the context effects. Your switchboard analysis is tackling this issue, but maybe it is worth emphasizing the necessary inclusion of gaze over decay to allow the decoy effects to be captured.

We want to thank the reviewer for raising the topic of the models’ accumulation leak.

While the leak included in the GLA indeed does not depend on gaze allocation, it still serves an important function in the model, as it allows the model to use choice-relevant information contained in the temporal order of fixations. Therefore, even though the leak itself does not depend on gaze, it has a vital gaze-dependent function when used in conjunction with other gaze-dependent mechanisms like the gaze-discount. In this sense, gaze-discount *and* leak are indeed both important components of the winning GLA model.

Note, however, that MDFT includes a leak term, but no gaze dependence. Yet, MDFT performs worse, so a simple leak does not suffice to perform well.

To illustrate and better communicate these implications to the reader, we have included additional wording in the main manuscript (ll. 382ff) and added a supplementary figure (Supplementary Fig. 4) and note (Supplementary Note 2). There, we describe in detail the interaction effects between gaze discount and accumulation leak mechanisms of GLA using model simulations.

6. It’s very interesting how the hybrid model combination from the switchboard can explain participants with higher RSTs. To stress this point, maybe is a good idea to include the average RST for the participants in each model category in table 2.

We thank the reviewer for this great suggestion. We included attraction and compromise RST in Table 2 (l. 578).

### Additional comments:

1. Line 728 : the title is repeated (“Gaze-biased leaky accumulator model (GLA)”)

- We addressed this error.

2. Line 557: “Additional mechanisms: For example, loss aversion”, capital F typo.

- Thank you for pointing this out. We fixed this error.

3. It may be a good idea to include the meaning of the acronyms for the model names in figure 3 legend.

- Thank you for the suggestion. We included the models’ names in the figure caption.

## Reviewer #2

### General remarks

In this manuscript, Molter et al. examined whether gaze-dependent evidence accumulation could explain context dependent effects in decisions under risk. First, they successfully elicited the attraction and compromise effects in their sample. Second, they fitted a number of computational models to the data. Models that did not take eye movements into account (EU & MDFT) provided a poorer fit to the data than models that did. In particular, the model that provided the best fit was a Gaze-dependent Leaky Accumulator (GLA), which assumed a multiplicative leaky integration of subjective amounts and probabilities. Then, the authors used the switchboard analysis to identify the contribution of specific model components. They found that the best model variant was equivalent to the GLA (provided the best for 17 subjects out of 40). Notably, a hybrid variant that in addition to gaze-dependent evidence accumulation also included distance dependent inhibition and comparative accumulation (as in the MDFT), provided the best fit for 9 subjects (especially those who showed strong attraction effect). In general, the article is clearly written and timely, the findings are innovative and show that in most cases, context effects can be explained using eye movements (without the need for specific mechanisms), and the statistical analyzes and computational models are rigorous and carefully executed. I have a few comments that I hope will help to improve the manuscript:

### Main points

1. In the introduction the authors mention the 3 context effects (attraction, compromise and similarity), however they focused only on two of them (compromise and attraction). What was the motivation for that?

We thank the reviewer for pointing us to this disparity. We have added clarifications for focusing on attraction and compromise effects in the introduction (ll. 101ff) and results (ll. 166ff) sections.

2. The authors examined two models that did not take eye movements into account (EU & MDFT). I think that they should also add the CPT.

We want to thank the reviewer for raising this point. We agree that Prospect Theory (PT) is an important theory to include in the model comparison, especially with the GLA using subjective valuation procedures like PT. We therefore included PT into our model comparison. Please note that, due to our gambles only having two outcomes, Cumulative Prospect Theory reduces to regular PT. We find that including PT does not change our results in any meaningful way.

In the process of adding PT to the model space, we additionally ran parameter- and model-recovery analyses which revealed issues in separating EU and PT (Supplemental Fig. 7). We think that this is not too surprising given that the experiment did not involve losses, only single outcomes, and no extreme probability values, all factors that would allow PT predictions to meaningfully differ from EU. Given that GLA uses PT-based inputs, we decided to keep PT as a reference model and remove EU from our analyses, resulting in better model recovery (Supplementary Fig. 8).

We have made corresponding changes to the manuscript, replacing EU with PT (ll. 395ff, ll. 936ff).

In addition, we outlined our parameter- and model-recovery procedures in a new methods section (ll. 1065).

a. Figure 3A shows the paradigm used in the study. In my opinion, the figure will be clearer if another panel, illustrating the compromise effect, will be added.

We thank the reviewer for this suggestion. We have added a panel showing a compromise trial and included additional panels to relate the paradigm panels to the attribute space.

b. I think that the figure will also be clearer if the authors mention in the caption that the location of the amounts and probabilities were randomly alternate on each trial.

We agree that this information helps clarity and added corresponding wording to the figure caption (ll. 135ff).

4. Glickman et al. fitted the GLA model using fixations as accumulation unit, and found that this provided a slightly better fit than using time intervals (e.g., 1ms) as accumulation unit. Did the authors attempt to make this comparison? In this context, do the findings presented in Fig. 5c&f remain the same if instead of relative dwell time, the DV is the relative number of fixations?

We thank the reviewer for this question. We have tested an alternative model variant that is based on fixation durations (quantized to 10 ms steps) and compared it against the originally included variant. In line with Glickman et al. (2019) we found this variant to perform worse for 29 of 40 participants.

We have added a paragraph on this in the results section (ll. 428ff) and a supplementary figure that replicates main manuscript figure 3 with this duration-based GLA variant (Supplementary Fig. 9).

5. Can the authors provide an intuition why the compromise effect was weaker in trial type CB than in trial type CA?

We included a section about possible reasons for why observed compromise effects were asymmetric with respect to the decoy type (ll. 250ff). We suspect that the indifference estimation procedure could be one reason for the observed asymmetries.

We also included a more elaborate discussion about the weaker compromise effects in our study (ll. 678ff), relating our results to marginal results from other studies and possible explanations.

6. Can the authors explain how the sample size was determined?

We thank the reviewer for raising this point. We determined sample size based on prior related work. We added a corresponding sentence to the methods section (ll. 842f).

7. The MDFT model predicts that the magnitude of context effects should increase with deliberation time. Is it the case in the data? (especially for the subjects who showed a strong attraction effect).

We thank the reviewer for raising the important issue of response times. We performed additional analyses of the association between response time and context effect strength on the group- and individual levels. We found no such association across participants (see Supplemental Fig. 1).

There was, however, a marginal effect within participants, such that participants were more likely to choose the target in compromise trials with slower response times. We added a section describing these analyses and results (ll. 265ff).

In addition, we added a paragraph to the discussion to disclose that our modelling approach did not account for response times more directly (ll. 821ff).

## Reviewer #3

### General remarks

In their paper, Molter and colleagues combine eye tracking with computational modeling to investigate two prominent context effects, the attraction and compromise effects, in risky choice. They find significant/credible evidence for the attraction effect but not for the compromise effect (though the trends are going in the predicted direction). In both situations, they also find that people look more at targets than competitors (and least at decoys). They go on to model the data in two steps: First they compare different established models that include or do not include eye movements to improve predictions, and find that a gaze-dependent LCA model (GLA) fits best. Then they use a “switchboard” approach to identify which mechanisms contribute most to predicting the data. This analysis again finds that the GLA fits best, but for a minority of participants with a strong attraction effect a model that assumes distance-dependent inhibition and comparative evidence comparison (much like Multi-alternative Decision Field Theory, MDFT) provides the best account.

Overall, the paper is well written and, in my view, relatively easy to follow, even though it covers and brings together several comparatively complicated topics (context effects in multi-alternative choice, sequential sampling models, eye tracking, advanced cognitive modeling). Some of the results may appear a bit weak (e.g., the absence of a compromise effect, and the fact that the switchboard analysis doesn’t really reveal that additional mechanisms beyond the GLA are critical to accommodate the behavior in the current task). However, it is more important to judge papers by the timeliness of their research question and the rigorousness of their methods, and I am very positive w.r.t. both of the aspects regarding the present paper. So far, there are only few papers that have looked at context effects with eye tracking (the paper cites two of them: Marini et al. and Noguchi & Stewart), and none of them have combined these topics with sophisticated state-of-the-art cognitive modeling techniques. Thus, I am quite enthusiastic about this paper but have some suggestions for corrections and additions that should be addressed in a revision.

### Major comments:

1. In the end, the winning model of this paper is basically the GLA. However, it is obvious that this model doesn’t really explain context effects. It is compatible with them as long as eye movements are in line with them (i.e., more fixations on targets than competitors), but on a mechanistic level, there is no explanation. The GLA does not predict that there will be more target-fixations (it just picks them up if this is how the data looks), and it does not assume a mechanism such as distance-dependent inhibition that would be able to predict context effects in some other (gaze-independent) way. I think this issue should be made more explicit in the discussion of the paper to be more clear about the limitations of GLA.

Relatedly, it should also be avoided that the paper gives the impression that context effects are solely driven by eye movements. I don’t think the authors want to convey such a message (and this is probably why they added the analyses around MDFT and the hybrid model), but again it would be good to be explicit here.

We thank the reviewer for raising this important issue. We agree, that the gaze-dependent model does not deliver a novel *explanation* of context effects in itself, but rather highlights a new perspective on the topic and links to a disconnected but influential field of work (namely gaze-dependent evidence accumulation models).

We have added a paragraph to the discussion, explicitly addressing this limitation (ll. 810ff) and changed wording elsewhere in the manuscript to communicate this more clearly.

2. A second limitation of this work (and the GLA, at least in the way it is used here) is that the question of response times (RT) is not addressed at all. Even though the models are dynamic (sequential sampling) models that would be able to predict RT, they are fitted to choices only. This is because the RT (and the eye movements) are used more in the form of inputs (information that helps the model to make better predictions) rather than as outputs / dependent variables. All of this is fine in the context of the present work, but it should be made explicit that the issue of predicting RT is not addressed here.

We thank the reviewer for also pointing us to this important caveat. In addition to additional analyses of response time and context-effect strengths (ll. 265ff and Supplemental Fig. 1), we have added a paragraph to the discussion, outlining this limitation, and suggesting future ways to address it (ll. 821ff).

Relatedly, models such as expected utility theory and MDFT have a disadvantage over models such as GLA, simply because they cannot leverage potential information that RT/eye-tracking data may contain. Thus, the first model comparison in the paper is somewhat unbalanced. This becomes most evident in the sentence starting in line 313 (page 17) “However, MDFT and EU generally could not capture the empirical association of gaze and choice;[…]”. Of course, they could not, because they do not make use of eye-tracking data. Theoretically, they could do so only if there is some third (hidden) factor that strongly ties eye movements to some element that these models do take into account.

We want to thank the reviewer for sharing their view on the model comparison. We agree, that the comparison is unbalanced with regard to the models’ use of process data, but note that this also deliberately one main point of our approach: Demonstrating the additional value of including process data into models, and evaluating models using those data.

However, we fully agree that this should have been made clearer in the manuscript and therefore qualified our model-comparison results to better reflect this inherent imbalance (ll. 493).

We also took this opportunity to motivate our following switchboard analyses more explicitly since it also aims to “level the playing field” between different model classes.

Speaking of: the small positive relationship between dwell time and choice probability that EU and MDFT seem to predict (see Fig. 3 c,d,e,f) is probably due to people looking more at high-value options (i.e., value-based attention; cf. Gluth et al., 2018/2020). This should be mentioned (there seems to be some evidence for it in Suppl. Fig. 1).

We thank the reviewer for highlighting this lack of discussion. We have added text to mention the gaze-independent models’ small predicted association between gaze and choice, and the potential source of this effect (ll. 474ff).

3. Also concerning the discussion, I think the two above-mentioned studies that have looked at the interplay of eye movements and context effects (Marini et al.; Noguchi & Stewart) warrant a more elaborate discussion. This may even be addressed earlier (in the introduction) to make clear what the novel contribution of the current paper over and above this previous work is supposed to be.

We thank the reviewer for pointing this out and suggesting improvements so clearly.

We have clarified our paper’s relationship to those two earlier studies in the introduction (ll. 89ff) and referred to their studies results in the discussion more explicitly (ll. 814ff)

I would also be interested in whether the authors can replicate the effects reported by Marini et al. and discussed on page 28 (fixations are shifted towards the target’s dominant attribute, and there are more target-decoy transitions).

We have also performed additional analyses replicating one of Marini et al.’s findings, namely the increased number of transitions between target and decoy in attraction trials. We have added this to the results section (ll. 319ff) and Supplementary Note 1

Unfortunately, our task setup did not allow for a test of the shift towards the target’s dominant alternative, since we only used range-frequency decoys, so the target dominates the decoy on *both* attributes.

4. The authors mention (page 17) that the gaze-dependent models appear to predict choices of dominated decoys in attraction trials if they have been looked at a lot. I was wondering, whether it could help to rescale/normalize the inputs to the model, so that comparatively low probabilities/magnitudes enter the models as negative values (or values very close to 0). In that case, looking at the decoy’s attributes could pull one away from this option.

:

We thank the reviewer for this concrete suggestion. Unfortunately, we have tried multiple versions of similar ideas to no avail. We see different difficulties in these approaches:

1. Normalisation of probability values (so that negative values ensue) is not compatible with multiplicative integration, which the GLA model relies on.
2. Dominated decoys are not aversive on their own. Even something like divisive normalisation of attributes does not yield aversive values for them. Their aversive character only results from attribute-wise comparisons to the target alternative. Such a mechanism, however, is very far from the GLA model (which was derived from prior work).

We note, however, that our switchboard analysis *did* include a variant that performed attribute-wise comparisons of the normalised attribute (additive integration included basic divisive normalization; attribute-wise gaze-discount could fully reduce comparisons to single attribute dimensions at every point in time; comparative processing performed item-vs-mean comparisons), yet this variant did not perform well.

If the reviewer has any specific implementation in mind, we would be happy to implement and include it in our analyses.

5. Given that GLA uses Prospect Theory-based values as inputs, it would be important to add Prospect Theory as a non-gaze comparison model in addition to EU and MDFT.

We want to thank the reviewer for raising this point. We agree that Prospect Theory (PT) is an important theory to include in the model comparison, especially with the GLA using subjective valuation procedures like PT. We therefore included PT into our model comparison. Please note that, due to our gambles only having two outcomes, Cumulative Prospect Theory reduces to regular PT. We find that including PT does not change our results in any meaningful way.

In the process of adding PT to the model space, we additionally ran parameter- and model-recovery analyses which revealed issues in separating EU and PT (Supplemental Fig. 7). We think that this is not too surprising given that the experiment did not involve losses, only single outcomes, and no extreme probability values, all factors that would allow PT predictions to meaningfully differ from EU. Given that GLA uses PT-based inputs, we decided to keep PT as a reference model and remove EU from our analyses, resulting in better model recovery (Supplementary Fig. 8).

We have made corresponding changes to the manuscript, replacing EU with PT (ll. 395ff, ll. 936ff).

In addition, we outlined our parameter- and model-recovery procedures in a new methods section (ll. 1065).

### Minor comments (not ordered by relevance):

6. Figure 1: in the figure, there probability difference between the decoy and the target is 3%, but in the legend, it says that the difference was always 2%. Please adjust / make consistent (whatever is correct, the figure or the text).

We thank the reviewer for highlighting this inconsistency. The difference was 2%. We have fixed the wrong value in the figure.

7. (I think) I can understand why the authors focused on 2 of the “big 3” context effects and left out the similarity effect (cf. Spektor et al., 2021, Trends Cogn Sci, for some discussion on why the similarity effect is rather distinct from the other two). However, it would again be helpful to be explicit here: what went into this decision?

We thank the reviewer for raising this point. We have added clarifications for focusing on attraction and compromise effects in the introduction (ll. 101ff) and results (ll. 166ff) sections.

8. Lines 131/page 7: “showing randomly created options with expected value of 10 EUR”. Was the expected value really 10, so for instance, in case of 5% probability, the amount shown on the screen would be 200? Also, I do not understand this notation: “(-3%, 0%, 3%)”. If this refers to the lower and upper end of a uniform distribution, what is the middle value “0%” meant to be?

We thank the reviewer for pointing us to this imprecise description of the stimuli and apologize for the confusion.

Filler trials indeed had options with expected values of 10, but the range of actual probability values used differed from the one reported in the previous version of our manuscript.

Regarding the noise that was added to increase variability between trials, options received either -3% or +3% probability, and -1 or ±0 (no change) EUR in outcome. We have amended the sections describing these details (ll. 189ff, 896ff).

9. Line 173/page 10: “did not exclude”. I’d suggest to change this double negative into “included”.

We thank the reviewer for this point and have followed their suggestion.

10. I would be curious to know what the authors’ thoughts are about the presence of a rather strong compromise effect in the eye movements but not in the behavior.

We think that this non-correspondence of gaze allocation and choice might be due to different factors:

One possible reason would be that our indifference estimation procedure did not result in full pairwise (and transitive) indifference between the set of alternatives.

In this case, even though gaze was allocated in favour of the compromise alternative, choices could still be driven to a stronger extent by the difference in subjective expected value. This highlights the modulatory role of gaze in the models: Its influence is combined with the alternatives’ underlying value estimates.

Notably, the asymmetry in compromise effect strength across target alternatives could be indicative of imperfect indifference estimation. We have included this reasoning into the discussion of weaker (ll. 678ff) and asymmetrical compromise effects (ll. 250ff).

Another possibility is that other eye movement parameters than relative dwell time, like the (relative) fixation count, are more coherently associated with choice behaviour (note that GLA, for example, uses fixations rather than their durations. A variant based on fixation duration, however, performed worse for 29 of 40 participants (Supplementary Fig. 9)

Finally, it is of course also possible that the association of gaze and choice is moderated by other, unknown factors.

We want to note, however, that models like the GLA are able to account for such non-correspondences, by reducing the gaze discount and leak strengths

11. Line 349 / page 19: typo: “differed” rather than “different”

Thank you for pointing this out. We have fixed this typo.

12. Figure 4c. It would be good to also highlight the Hybrid model, e.g., with a differently-colored frame.

We are thankful for this suggestion. We have included a dashed frame around the hybrid variant in Figure 4c.

13. Line 486/page 28: “providing novel evidence for the compromise effect in risky choice”. I would lower the tone here, because it sounds as if the study found significant/credible effects for it.

We agree with the reviewer that this wording was too strong and have toned it down accordingly. In addition, we included some discussion of the weaker compromise effects in this study (ll. 678ff)

14. Participants: I always ask authors to justify their sample size, whether it’s a based on a formal power analysis, previous related work, or just experience.

We thank the reviewer for raising this point. We determined sample size based on prior related work. We added a corresponding sentence to the methods section (ll. 842f).

15. Line 634/page 34: typo: “mi”; the i should be subscripted.

Thank you for pointing out this error. We have fixed it.

16. Line 43/page 3: “and others that apply explicitly to”. I’d suggest to change to “and others that are applied explicitly to”.

We have followed the reviewer’s suggestion for improved clarity.
