# Supplementary material for: Gaze-dependent evidence accumulation predicts multi-alternative risky choice behaviour
Source: PLoS Comput Biol. 2022 Jul 6;18(7):e1010283. doi: 10.1371/journal.pcbi.1010283 (PMC9292127; doi:10.1371/journal.pcbi.1010283)
Supplement: Attachment — Submitted filename: molter_et-al_gda-context_point-by-point-response_revision-2.docx [file pcbi.1010283.s023.docx]

# Point-by-point-response

## Reviewer 1

I want to thank the authors for addressing my points. I am satisfied with their answers and modifications. I am making some final extra suggestions just to clarify some points in the latest version:

1. Line 758: Since it is included in the discussion that GLA model does not explain fixation allocation, it may be useful to also mention these recent papers that explore policy and optimality of gaze allocation during decisions: Jang, Sharma and Drugowitsch, eLife, 2021; and Callaway, Rangel, Griffiths, PlosCompBiol, 2021. Of special interest may be Callaway et al. since it also models trinary choices.

We thank the reviewer for this suggestion. We agree that these two recent references should be mentioned, as they directly address one limitation of our work. We have revised the section on other existing work exploring generative models of the fixation process and explicitly mentioned these two studies’ focus on optimality:

**ll. 777ff:**

“A promising but technically and theoretically daunting way to address these limitations is the inclusion of generative models of the fixation process into gaze-dependent decision-making models. ~~While t~~This approach was successful in some specific choice settings^2,6,11,43,62^, with two recent studies investigating the optimal policy of allocating visual attention given a gaze-dependent decision mechanism and finding empirical data to match the optimal policy in simple two- and three-alternative choice^75,76^. Extensions to settings with multiple visually presented alternatives and attributes, however, remain a challenge to be addressed in future work.”

2. Line 685: it is not clear to me what is referring “this way” in that sentence.

We thank the reviewer for pointing out this poorly phrased sentence. We have removed the unnecessary "this way" to increase clarity:

**ll. 692f:**

“While our results do not provide a full explanation of context-dependent choice behaviour ~~this way~~ (see below), they still suggest visual attention as a potential mediator.”

3. Line 832: not sure what ± 0 means.

We thank the reviewer for highlighting this. We have revised our notation was not clear and have therefore revised it to be more explicit:

**l. 847:**

“To reduce repetition of stimulus values, for each trial, noise was added to each option’s outcome *m* (-1 EUR or no change) […]”

## Reviewer #2

The authors have well addressed my previous comments in the revision. A few more minor comments:

1. I think that the authors should mention in the text that for the lotteries which were used, the predictions of the PT & CPT are equivalent.

We thank the reviewer for this comment. We have added this detail to our results:

**ll. 376ff:**

“First, we included Prospect Theory, which assumes the same alternative-wise valuation as GLA, but is agnostic to gaze data. Note that for the all-or-nothing gambles used in our experiment, predictions of Prospect Theory and Cumulative Prospect Theory are identical.“

2. Line 238 – "Finally, it remains possible, that participants’ preferences shifted towards higher outcomes after the estimation block, leading to stronger preferences for the CB decoy and reduced compromise effects in this condition.". This explanation seems quite plausible; Do the data of the other conditions (e.g., attraction trials/fillers) support it?

We thank the reviewer for this additional suggestion. Looking at mean choice probabilities, we do not find a general trend towards high-outcome alternatives across experimental conditions (see Table 1) or in the filler trials. We have added this qualification to the end of the respective paragraph:

**ll. 240ff:**

“Finally, it remains possible, that participants’ preferences shifted towards higher outcomes after the estimation block, leading to stronger preferences for the CB decoy and reduced compromise effects in this condition. However, preference for higher outcomes was only observed in compromise and attraction trials targeting alternative B (Table 1). Other alternatives were preferred in the remaining conditions, and overall participants chose the highest outcome only in 38% of the trials (in distractor trials, the highest outcome was only chosen in 21% of trials).”

3. Line 91 – et. al. should be replaced with et al (the period should be removed).

We thank the reviewer for highlighting this error. We have removed the period:

**ll. 91ff:**

“Similarly, Marini et~~.~~ al. found that adding an asymmetrically dominated decoy to a choice set shifts gaze towards the target’s dominant attribute and leads decision makers to make more transitions between target and decoy alternatives.”

4. Supplementary figure 4 – I think it would help to indicate that the dashed horizontal line represents chance level, and to add a reference to Supplementary note 2 in the caption of the figure.

We thank the reviewer for these suggestions. We fully agree, that both suggestions will help readers follow the simulation procedure and its results and have changed the manuscript accordingly:

**Supplementary Information, ll. 97f:**

“All predictions are based on mean empirically estimated model parameters (except for leak parameters λ and the discount parameter θ in b) of trials with three alternatives with equal expected subjective value and all possible sequences of 6 fixations. Horizontal dashed lines represent chance level. See Supplementary Note 2 for details on the simulation analysis.”
